# Transglutaminase 2 Up-Regulation Is Associated with Inflammatory Response in PBMC from Healthy Subjects with Hypovitaminosis D

**DOI:** 10.3390/medsci6040103

**Published:** 2018-11-16

**Authors:** Daniela Caccamo, Nadia Ferlazzo, Monica Currò, Sergio Ricca, Riccardo Ientile

**Affiliations:** Department of Biomedical and Dental Sciences and Morphofunctional Imaging, University of Messina, Via Consolare Valeria, 98123 Messina, Italy; dcaccamo@unime.it (D.C.); nadiaferlazzo@email.it (N.F.); moncurro@unime.it (M.C.); ricca.sergio85@yahoo.com (S.R.)

**Keywords:** immune response, NF-κB, peripheral blood mononuclear cells, transglutaminase 2, vitamin D

## Abstract

Recent evidence indicated that transglutaminase 2 (TG2) is involved in the adaptive immune response. Peripheral blood mononuclear cells (PBMC) have largely been used to characterize molecular mechanisms occurring in the activation of immune response. Given that the maintenance of immune system functions requires an optimal vitamin D status, we aimed to assess the involvement of TG2/NF-κB signaling in cytokine production in PBMC isolated from adult subjects with different vitamin D status. We observed TG2 up-regulation and a significant positive correlation between TG2 expression and tumor necrosis factor (TNF)-α mRNA levels in PBMC of recruited patients. The mRNA levels of TG2 and TNF-α were higher in PBMC of subjects having hypovitaminosis D, namely plasma 25(OH)vitamin D3 levels lower than 50 nmol/L, than in those with normal vitamin D levels. Moreover, NF-κB up-regulation and nuclear translocation were detected, concomitantly with TG2 as well as TNF-α increased expression, in PBMC of vitamin D-deficient subjects. The present findings confirm that an increase in TG2 expression exacerbates the activation of NF-κB and the production of pro-inflammatory cytokines, and suggest a link between vitamin D deficiency, TG2 up-regulation, and inflammation.

## 1. Introduction

Tissue transglutaminase or transglutaminase 2 (TG2) is a unique member of the transglutaminase family since it displays various enzyme activities, acting as a transamidating enzyme, GTPase/ATPase, protein disulfide isomerase, protein kinase and has non-enzymatic functions based on its non-covalent interactions with multiple cellular proteins. Moreover, it is ubiquitously distributed and localized in different cell compartments and in the extracellular milieu [1,2].

Due to its multifunctional behavior, TG2 regulates a variety of cellular processes, such as growth, differentiation, survival, apoptosis, extracellular matrix organization, adhesion, and migration [3,4]. It plays a key role in various physiological responses and pathological conditions, i.e., wound healing, inflammation, cancer, and neurodegeneration [5].

Recent evidence demonstrated that TG2 expression is involved in the complex biological mechanisms associated with the adaptive immune response. Indeed, TG2 expression has been reported in peripheral blood mononuclear cells (PBMC), a subpopulation of white cells, consisting of 70% T lymphocytes, 15% monocytes, 10–15% natural killer cells, 5–10% B lymphocytes, and 0.5–1% dendritic cells [6]. Notably, PBMC have proven useful in several studies on the immune response activation, in regard to inflammation. The inflammatory response may be characterized through the assessment of the morphological and functional changes in most lymphocytes and PBMC, as well as altering circulating levels of inflammation biomarkers, such as interleukins, chemokines, and other signaling molecules [7,8]. Importantly, PBMC can be easily collected, representing a largely accessible material for studies aimed at characterizing molecular pathways involved in the activation of immune responses associated with different regulators [9].

Recent scientific evidence suggests that the maintenance of immune system functions requires an optimal vitamin D status, resulting from an adequate vitamin D dietary intake and intense outdoor activities [10]. In vivo and in vitro experiments demonstrated that vitamin D enhances the production of anti-inflammatory cytokines (transforming growth factor (TGF)β-1 and interleukin (IL)-4), and reduces the production of the pro-inflammatory ones (IL-6, interferon-gamma (IFN-γ), IL-2, and tumor necrosis factor (TNF)-α) [10,11]. Although the molecular mechanisms for vitamin D-dependent regulation of immune response are not fully clarified, accumulating pre-clinical and clinical evidence suggests a link between the immunological profile and activation of 1α,25-dihydroxyvitamin D3 signaling pathways, such as the nuclear factor-κB (NF-κB) system [12].

The NF-κB system comprises a family of ubiquitous, inducible transcription factors, primarily regulated by inhibitory I-κB proteins. A key event in the activation of the NF-κB pathway is the translocation of the NF-κB complex from the cytosolic compartment into the nucleus. The NF-κB signaling pathway is an important regulator of immune response and inflammation [13]. It has been shown that the activation of NF-κB, JAK/STAT, and MAPK signaling pathways is linked to the transcriptional up-regulation of cytokines, such as IL-6, IL-2, or IL-10 [14,15].

It is well known that NF-κB may be activated independently of I-κB kinase phosphorylation, through the TG2-mediated I-κBα cross-linking and polymerization. This alternative pathway particularly occurs in inflammatory conditions [16]. However, it is still unclear to what extent the immune response is dependent on cytokine expression induced by TG2/NF-κB signaling.

The aim of this study was to assess the involvement of TG2/NF-κB signaling in cytokine production, in PBMC isolated from subjects with different vitamin D statuses.

## 2. Materials and Methods

### 2.1. Study Cohort

Thirty-two male patients (mean age 59.2 ± 8.0) attending the diagnostic laboratories in the Operative Unit of Clinical Biochemistry at Polyclinic Hospital University (Messina, Italy) were enrolled for this study.

Blood samples were collected in test tubes containing ethylenediaminetetra-acetic acid (EDTA) from all subjects. Informed written consent was obtained from all participants. The study was conducted in accordance with the Declaration of Helsinki, and the protocol was approved by the local ethics committee (University of Messina, ethical code 12/16–22/03/2016).

Plasma was obtained after blood centrifugation. Aliquots of whole blood and plasma were stored at −20 °C until analysis.

### 2.2. Assessment of Vitamin D Serum Levels

The quantitative determination of 25(OH)Vitamin D3 plasma levels was performed by high-performance liquid chromatography (HPLC) with a Bio-Rad 25-OH Vitamin D3/D2 kit.

### 2.3. Isolation of Peripheral Blood Mononuclear Cells

Peripheral blood mononuclear cells were isolated by centrifugation on a Ficoll-Histopaque density gradient. In brief, the blood collected was diluted 1:2 in phosphate buffer (PBS), layered on Ficoll, and centrifuged at 400× *g* for 20 m. Peripheral blood mononuclear cells, appearing as a concentrated band in the Ficoll-plasma interface, were harvested, washed twice with PBS, and stored at −80 °C until further analysis.

### 2.4. Quantitative Real-Time PCR Analyses

Total RNA isolation was carried out using the TRIzol reagent (Invitrogen, Milan, Italy) according to the manufacturer’s instructions. Two micrograms of total RNA were reverse transcribed into cDNA by using the High-Capacity cDNA Archive Kit. Then, mRNA levels of TG2, NF-κB p50 subunits, NF-κB p65 subunits, and TNF-α were assessed by quantitative real-time PCR using SYBR Green-based gene expression analysis. β-actin was used as endogenous control. The primer sequences used are listed in Table 1. Quantitative PCR reactions were carried out in 20 µL reactions containing 1x SYBR green PCR Mastermix, 0.1 µM specific primers, and 25 ng RNA converted into cDNA. Real-Time PCR was performed in a 7900HT Fast Real-Time PCR System (Applied Biosystems, Foster City, CA, USA) with the following profile: one cycle at 95 °C for 10 m, followed by 40 cycles at 95 °C for 15 s and 60 °C for 1 m. For SYBR green assays a standard dissociation stage was added to assess primer specificity. Data were collected with SDS 2.3 software (Applied Biosystems, Foster City, CA, USA) and analyzed using the 2 ^−ΔΔCt^ relative quantification method.

### 2.5. Western Blotting

To obtain whole cell extracts, cells were lysed using ice-cold RIPA buffer supplemented with Protease Inhibitor Cocktail (SIGMA Aldrich, Milan, Italy), and cell debris was removed by centrifugation at 8000× *g* at 4 °C for 10 m. Protein concentration was evaluated by the Bradford method, and 30 µg proteins were loaded on a 10% denaturing SDS-polyacrylamide gel, and transferred to a nitrocellulose membrane. After protein transfer, the membranes were blocked with 5% non-fat dry milk at room temperature for one hour. Detection of specific proteins was performed by probing membranes with mouse monoclonal antibodies against TG2 and β-actin (1:1000 and 1:5000 in TBS-T, respectively) overnight at 4 °C, and subsequently incubating them with horseradish peroxidase-conjugated anti-mouse secondary antibody (diluted 1:5000 in TBS-T) for 2 h at room temperature. Immunoblots were developed with ECL Plus chemiluminescent detection system kit (Pierce, ThermoFisher, Monza, Italy) using Kodak film. The bands were scanned and quantified by densitometric analysis with an AlphaImager 1200 System (Alpha Innotech, San Leandro, CA, USA).

### 2.6. Electrophoretic Mobility Shift Assay

Nuclear proteins were isolated as described by Caccamo et al. [17]. After quantification, nuclear proteins (2 μg) were incubated with the double-stranded NF-κB probe, that had previously been biotin-end labeled by terminal deoxynucleotidyl transferase included in the Biotin 3′ End DNA Labeling Kit (Pierce, ThermoFisher, Monza, Italy).

Protein/DNA complexes were resolved by electrophoresis on a non-denaturing 6% polyacrylamide gel. After electroblotting onto a nylon membrane, detection of protein/DNA complexes was performed using chemiluminescence methods using streptavidin-HRP.

Bands were scanned and quantified by densitometric analysis with ImageJ 1.47 (National Institutes of Health, Bethesda, MD, USA).

### 2.7. Statistical Analysis

All values are expressed as mean ± standard deviation (SD). Statistical analysis was carried out using Student’s *t* test for comparisons between two groups. A *p* value lower than 0.05 was considered significant. Correlation analysis was used to describe the relationship between TG2 and TNFα levels.

## 3. Results

We first assessed vitamin D3 serum levels in our study cohort. More than 50% of recruited subjects (*n* = 18) had normal vitamin D3 levels (77.1 ± 16.8 nmol/L), while the remaining 14 subjects exhibited a status of hypovitaminosis D, namely vitamin D3 levels lower than 50 nmol/L [18] (26.7 ± 11.4 nmol/L). As a consequence, the recruited subjects were divided in two subgroups, one including individuals having normal vitamin D3 levels (>50 nmol/L) and the other including individuals having deficient vitamin D3 levels (<50 nmol/L).

**Figure 1:**
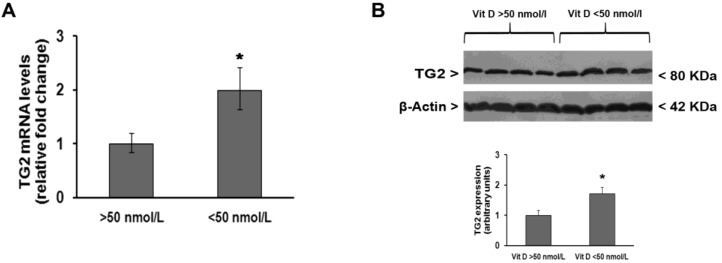
Changes in transglutaminase 2 (TG2) expression in peripheral blood mononuclear cells (PBMC) are influenced by vitamin D status of adult male individuals recruited for this study. (**A**) Quantitative Real-time PCR analysis of TG2 mRNA levels after normalization against β-actin mRNA levels; (**B**) representative immunoblot of TG2 protein levels and densitometric analysis of TG2 immunoreactive bands after normalization against β-actin levels. * *p* < 0.05, significant difference in comparison to individuals having normal vitamin D levels.

The analysis of TG2 expression in PBMC isolated from all recruited subjects showed that TG2 mRNA transcript levels were significantly lower in subjects with normal vitamin D3 levels than in those with hypovitaminosis D (Figure 1A). These observations were confirmed by Western blot analysis of TG2 protein levels (Figure 1B).

**Figure 2:**
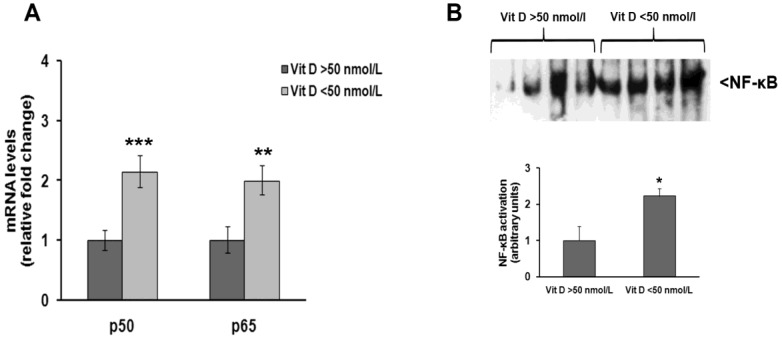
NF-κB expression and activation are increased in PBMC of vitamin D deficient individuals recruited for this study. (**A**) Quantitative Real-time PCR analysis of NF-κB p50 and p65 subunits mRNA levels after normalization against β-actin mRNA levels; (**B**) representative electrophoretic mobility shift assay (EMSA) pictures of active NF-κB nuclear protein levels and densitometric analysis of NF-κB bands. * *p* < 0.05, ** *p* < 0.01, *** *p* < 0.001, significant differences in comparison to individuals having normal vitamin D levels.

Quantitative real-time PCR analysis also showed that NF-κB p50 and p65 mRNA levels were two-fold higher in subjects with vitamin D3 deficiency than in those with normal vitamin D3 levels (Figure 2A), and this difference was found to be statistically significant. Electrophoretic mobility shift assay (EMSA) analysis of nuclear proteins in both groups confirmed real-time PCR results, showing that NF-κB was activated to a greater extent in subjects with hypovitaminosis D than in those with normal vitamin D3 levels (Figure 2B).

**Figure 3:**
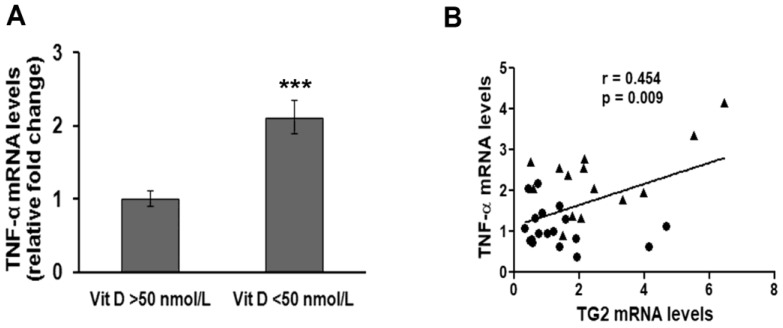
Assessment of TNF-α mRNA levels and correlation with TG2 mRNA levels in PBMC of individuals recruited for this study. (**A**) Quantitative Real-time PCR analysis of TNF-α mRNA levels after normalization against β-actin mRNA levels. *** *p* < 0.001, significant difference in comparison to individuals having normal vitamin D levels. (**B**) Plot showing results of correlation analysis between TG2 and TNF-α mRNA levels in the study cohort. ● subjects having normal vitamin D levels; ▲ subjects having deficient vitamin D levels. *p* = 0.009, statistically significant difference according to a *p* value <0.05.

In order to understand whether TG2 up-regulation and NF-κB activation were accompanied by the activation of inflammation pathways, we also assessed the mRNA levels of the pro-inflammatory cytokine TNF-α. We found that people with deficient vitamin D3 levels had a two-fold increased TNF-α expression compared to people with normal vitamin D3 levels, and this difference was statistically significant (Figure 3A). Notably, a highly significant, positive correlation was found between normalized TNF-α mRNA levels and TG2 mRNA levels (Figure 3B).

## 4. Discussion

Peripheral blood mononuclear cells are important components of the immune system, that constantly interact with other cells and are therefore relevant for studying the inflammatory process [7]. Local and systemic inflammation is the body’s response to tissue injury and involves the release of both pro- and anti-inflammatory cytokines. The adaptation to environmental inflammatory stimuli always requires the regulation of gene expression. Thus, molecular mechanisms that reflect the early stages of activation in the immune system can be studied through gene expression analyses. Therefore, the assessment of mRNA transcript levels may represent a sensitive tool in characterizing the effects of inflammatory stimuli on the immune system early [7].

In this study, we demonstrated the up-regulation of TG2 and a significant positive correlation between TG2 expression and TNF-α mRNA levels, in PBMC of adult patients. These results are in agreement with previous studies showing that the expression of TG2 in PBMC is associated with an inflammatory response [19,20]. In particular, several studies have emphasized the involvement of TG2 in the initial phase of cell damage and inflammation. Indeed, the cytokines and growth factors secreted during the early stages of cell injury regulate the expression of TG2 [21]. Recently, we demonstrated the up-regulation of some inflammatory markers, such as TNF-α, IL-6, and high mobility group box 1 (HMGB1), and at the same time the increase of TG2 mRNA levels; in human periodontal ligament cells obtained from patients with chronic periodontitis, compared with healthy subjects [22]. Interestingly, high-molecular weight and denaturing-resistant HMGB1 complexes, rising from protein-cross-linking activity of TG2 enzyme, have been observed in the plasma and PBMC of individuals with immune disorders and, to a much lesser extent, in healthy subjects [23].

In this study, high mRNA levels of TG2 and TNF-α were found in subjects with hypovitaminosis D, namely serum 25(OH)vitamin D3 levels lower than 50 nmol/L, according to the estimated current cut-off value [24]. Indeed, serum 25(OH)vitamin D3 levels have been regarded as a reliable biomarker for the assessment of vitamin D status [25].

The role of vitamin D3 in immunomodulation has been highlighted following epidemiological observations, that show an increased susceptibility to pathogen infections in vitamin D-deficient individuals, and claim an effective prevention of infectious diseases by vitamin D supplementation [26]. The biological activities of vitamin D are mediated by the interaction with the vitamin D receptor (VDR), a specific intracellular receptor with selective action, belonging to the superfamily of steroid hormone receptors. Notably, the majority of immune cells, i.e., CD4+ and CD8+ T lymphocytes, B lymphocytes, neutrophils, macrophages, and dendritic cells, express the VDR. Notably, VDR expression is further increased in the presence of pathogenic stimuli. Moreover, the above mentioned cells possess the CYP27B1 enzyme, catalyzing the conversion of 25(OH)vitamin D3 in 1,25(OH)_2_vitamin D3, thus they are able to produce active vitamin D3.

Vitamin D was also shown to be crucial for normal macrophage functions, and its deficiency was related to impaired chemotaxis, phagocytosis [27], and up-regulation of monocyte toll-like receptors (TLRs) which are well-known inducers of inflammation [28]. During inflammation, immune cells activated by TLRs have the ability to undergo a bioenergetic switch towards glycolysis, paralleled by an increase in reactive oxygen species and lipid peroxidation, and a decrease in the mitochondrial membrane potential [29]. Notably, vitamin D deficiency has been associated with increased oxidative and glycolytic bioenergetic profile responses in PBMC obtained from adults [30].

Relevant features of vitamin D action are the cell-type specific transcriptional regulation of genes involved in the inflammatory response, and the interplay between vitamin D-activated pathways and other inflammatory signaling cascades [12]. For example, active transcriptional complexes built through the heterodimeric association of VDR with the retinoid X receptor (RXR) are able to suppress the transcription of genes encoding for inflammatory cytokines, through the replacement of nuclear factors in activated T cells [31]. Notably, an age-dependent, significant decrease in the expression of RXR beta subtype of retinoid receptors, likely reflecting physiological senescence, has been demonstrated in PBMC of healthy elderly men (65.4 +/− 3.8 years) [32]. These observations suggest that when vitamin D serum levels fall below the normal ranges, the VDR/RXR-mediated activation of anti-inflammatory pathways is strongly reduced, or even completely inhibited. In this regard, VDR-mediated suppression of RelB NF-κB subunit expression in antigen presenting cells [33], and the vitamin D3-induced inhibition of NF-κB signaling through the physical interaction of VDR with p65 subunit [34], have also been reported. The latter are both paradigms for ligand-augmented negative transcriptional regulation.

Notably, in this work, we observed NF-κB up-regulation and nuclear translocation in vitamin D deficient subjects. Changes in NF-κB expression and activation were concomitant with TG2, as well as increased TNF-α expression. Our findings are in agreement with a previous report showing that the overexpression of TG2 results in constitutive NF-κB activation in the presence of TNF-α [35].

In conclusion, the present observations confirm that the occurrence of an increase in TG2 expression exacerbates NF-κB activation in inflammatory states, and further suggests a link between vitamin D deficiency, TG2 up-regulation, and inflammation.

Further studies will be useful in elucidating the suppression mechanism of TG2 expression by vitamin D, i.e., using experimental models based on vitamin D supplementation.

## Figures and Tables

**Table 1:** Primer sequences used for quantitative real-time PCR analysis of mRNA transcript levels.

Gene	Primer	SequenzaPrimer 5′→3′
ACT-β	forward	TGGTTACAGGAAGTCCCTTGCC
ACT-β	reverse	ATGCTATCACCTCCCCTGTGTG
NF-κB p50	forward	ACACTGGAAGCACGAATGACAGA
NF-κB p50	reverse	CCTCCACCTTCTGCTTGCAA
NF-κB p65	forward	CAGGCGAGAGGAGCACAGATAC
NF-κB p65	reverse	TCCTTTCCTACAAGCTCGTGGG
TNF-α	forward	GTGAGGAGGACGAACATC
TNF-α	reverse	GAGCCAGAAGAGGTTGAG
TG2	forward	CCTTACGGAGTCCAACCTCA
TG2	reverse	CCGTCTTCTGCTCCTCAGTC
